# Evaluation of the proximate composition, antioxidant potential, and antimicrobial activity of mango seed kernel extracts

**DOI:** 10.1002/fsn3.399

**Published:** 2016-06-25

**Authors:** Jane K. Mutua, Samuel Imathiu, Willis Owino

**Affiliations:** ^1^ Department of Food Science and TechnologyJomo Kenyatta University of Agriculture and TechnologyP. O. Box 62000Nairobi00200Kenya

**Keywords:** Antimicrobial potential, antioxidants, mango kernels, polyphenols

## Abstract

After pulp extraction in fruit processing industry, a significant quantity of mango seed kernels are discarded as solid wastes. These seed kernels can be ideal raw materials for obtaining extracts rich in bioactive compounds with good antioxidant properties. The conversion of these wastes into utilizable food ingredients would help in reducing environmental problems associated with processing waste disposal. In order to determine their potential use, this study evaluated some of the biochemical characteristics and antimicrobial potential of mango seed kernel extracts on medically important human bacterial and fungal pathogens. Four mango varieties (Apple, Ngowe, Kent and Sabine) from Makueni and Embu counties in Kenya were used for this study. The analyzed mango seed kernel powders were found to contain on average, 6.74–9.20% protein content. Apple and Ngowe mango seed kernels had significantly higher fat content of 13.04 and 13.08, respectively, while Sabine from Makueni had the least fat content of 9.84%. The ash, fiber, and carbohydrate contents ranged from 1.78 to 2.87%, 2.64 to 3.71% and 72.86 to 75.92%, respectively. The mean percentage scavenging ability of mango kernel extracts at the concentration of 20 mg/mL was 92.22%. Apple and Sabine mango kernel extracts had significantly high inhibition zones of 1.93 and 1.73 compared to Kent and Ngowe with 1.13 and 1.10, respectively, against *E. coli*. For *C. albicans,* the inhibition of Kent mango kernel extract, 1.63, was significantly lower than that of Ngowe, Apple, and Sabine with 2.23, 2.13, and 1.83, respectively. This study demonstrates that mango seed powder is an abundant and cost‐effective potential natural antibiotic and antifungal that can be utilized in addressing the challenge of food poisoning and infections caused by pathogenic microorganisms in the food industry.

## Introduction

Mango fruit originated from South Asia from where it was dispersed to the rest of the world (Litz 2009). Currently, the mango fruit is grown in over 90 countries in the world, the Asian continent being the biggest mango producer accounting for almost 77% of the total world's production (FAOSTATS, 2010). In Kenya, for the past decade, mango production has been on the increase due to increased demand as a majority of the population appreciates its health benefits. In 2014, the area under mango in Kenya was 47,620 hectares producing 744 million tons valued at ~$US 101 Million (HCDA, 2014).Morphologically, the mango fruit is divided into the epicarp, mesocarp, and the endocarp. The fleshy part of the fruit, mesocarp, is utilized during processing of juice, pulp, and mango slices for canning. Although the size of the epicarp and endocarp are highly influenced by the varieties, they account for 15–20% and 20–60% w/w of the whole fruit, respectively, while the kernel accounts for 45–75% of the endocarp (Ashoush and Gadallah 2011). The proximate composition of the mango seed kernel is 2.58% ash, 11.55% fat, 6.02% protein, 10.60% crude fiber, and 59.34% carbohydrates, with a moisture content of 9.89% (Omotubga et al. 2012).

The waste generated from the mango processing industry, derived mainly from the epicarp and endocarp has been estimated at 75000mT (Dorta et al. 2012), and is on the rise due to growth in mango fruit production and processing industry. Solid food processing waste materials cause serious environmental problems, such as water pollution, unpleasant odors, asphyxiation, vegetation damage, and greenhouse gas emissions (Gupta et al. 2015). In addition, waste disposal is costly and adds to the total cost of production.

Emergence of bacterial multidrugs resistance phenomenon such as methicillin‐resistant *Staphylococcus aureas* (MRSA) is becoming a global concern hence necessitating the search for more and better antimicrobial agents (Nikaido 2009). Different plant parts which have been utilized as herbal remedies in the past have turned out to be good sources of antibiotics in the modern world (AEl‐Gied et al. 2012). These plant parts can be utilized to fight of food pathogens that lead to spoilage and cause food poisoning. Establishment of safe plant parts that can be utilized in food preservation can go a long way in promoting public health since these extracts are of natural origin. Antimicrobial properties of plant origin are usually attributed to the presence of bioactive substances such as flavonoids, polyphenols, and essential oils (Calvo et al. 2012). Phenolic compounds are some of the major natural antioxidants currently recognized, due to the presence of a large number of phytochemicals with a greater antioxidant capacity (Boskou 2006). Other than their antioxidant capability, they are also associated with treatment options of some of the chronic diseases such as diabetes, hypertension, and dyslipidemia (Mumper and Dai 2010).

Some of the common pathogens of public health concern are *Staphylococcus aureus*, pathogenic *Escherichia coli*, and *Candida albicans*. *Staphylococcus aureus* is a gram‐positive, nonmotile and nonspore‐forming bacterium which is a facultative anaerobe capable of generating energy by aerobic respiration yielding lactic acid (Konrad et al. 2009). *S. aureus* is an important pathogen whose significance has risen due to its increased antibiotic resistance. The bacterium is known to cause infections, such as pneumonia, meningitis, osteomyelitis, endocarditis, and toxic shock syndrome (Jiamboonsri et al., 2011). Blood *Escherichia coli* are gram‐negative rod‐shaped bacteria which are oxidase negative. They are facultative anaerobes and their presence in food is an indicator of fecal contamination. There are five key diarrheagenic *E. coli* pathogens namely, Shiga toxin‐producing *E. coli* (STEC), enteroaggregative *E. coli* (EAEC), diffusely adherent *E. coli* (DAEC), enteropathogenic *E. coli* (EPEC), enteroinvasive *E. coli* (EIEC), enterotoxigenic *E*. *coli* (ETEC), and adherent invasive *E. coli* (AIEC) (Croxen et al. 2013). The major clinical symptoms associated with these pathogens are; urinary tract infections (UTIs), enteric/diarrheal disease, and sepsis/meningitis (Kaper et al., 2004).

*Candida albicans* is an opportunistic human pathogen. It is the most predominant cause of fungal diseases such as thrush/esophageal candidiasis, vaginal yeast infections, and invasive candidiasis. In the USA, the *Candida* species are ranked fourth in causing nosocomial bloodstream infections with a mortality rate of 35% (Yun‐Liang 2003). It is easy to handle and contain *Candida albicans* than other pathological fungi of medical importance such as *Aspergillus fumigatus* and *Cryptococcus neoformans* (Kabir et al. 2012).The inadequate availability of antifungal drugs coupled with fungal resistance to the drugs already in the market calls for the introduction of more effective antifungal drugs.

There have been reports indicating high antioxidant capacity of solvent extracts from mango biowaste (Dorta et al. 2012). However, there are limited reports to our knowledge of the determination of the effects of these extracts on specific pathogens of public health concerns. This study was undertaken with the objective of evaluating the proximate composition, antioxidant capacity, total polyphenols, and antimicrobial potential of the mango seed kernel extracts on medically important human bacterial and fungal pathogens.

## Materials and Methods

### Study site and experimental materials

Four mango fruit varieties (Apple, Kent, Ngowe and Sabine) were obtained from farms in Makueni (37° 15′ 00″ E, 1° 12′ 00″ S) and Embu (37° 38′ 23″ E, 0° 34′ 35″ S) counties in Kenya. The fruits were obtained from farms with the specific varieties within these locations. The sample size was 1600 mangoes, 200 samples for each variety and from the different locations. Uniform maturity of the fruits was ensured by picking the fruits at stage four and five of maturity, characterized by full shoulders at the stem end. They were then transported in aerated plastic crates to the Jomo Kenyatta University of Agriculture and Technology (JKUAT) postharvest laboratory where they were washed thoroughly to remove any dirt. The clean mangoes were stored at room temperature until fully ripe. The chemicals used were from Sigma‐aldrich company and while the media were from Oxoid.

### Sample preparations

Mango fruits were peeled with a sharp knife and the flesh was separated from the seed using a blunt knife. The seeds were sundried for 2 days followed by oven drying at 70°C until they attained a constant weight. Kernels were then manually removed from the seeds, ground with a hammer mill (Type EFOU single phase induction motor, split phase, Tokyo, Japan), and the powder sieved through a 500 *μ*m diameter sieve. The powder was then stored in self‐sealed paper bags and kept under refrigeration at 4°C until utilization.

### Determination of proximate composition

Moisture, ash, crude fiber, and crude fat were determined according to the A.O.A.C (1995) method numbers 930.04, 923.05, 960.39, and 920.86, respectively, while carbohydrates were calculated by difference. Nitrogen content was determined by micro‐Kjeldahl method and the factor 6.25 was used to convert the nitrogen to protein method number 46‐13.01 (AACC, 1999 1999).

### Determination of antioxidant capacity

*Sample Extraction*: Sample extraction was done according to the method described by Bloor 2001. A half gram from each of the samples was extracted with 20 mL of methanol diluted with water in the ratio 3:2 v/v. The mixture was centrifuged at 700g for 10 min at room temperature. The supernatant obtained was adjusted to 25 mL in a volumetric flask. An aliquot of this extract was used for the quantification of total phenolic and antioxidant activity.

The ability of the extracts to scavenge DPPH free radical was determined by the method described by Blois (1958). Two milliliters of the mango kernel powders extract were mixed with 10 mls of 0.1 mmol/L DPPH in methanol. The mixtures were allowed to stand for 20 min after which their absorbance was taken at 517 nm against methanol blanks using UV‐1800 spectrophotometer (Shimadzu, Kyoto, Japan). The percentage scavenging effect was calculated from the decrease in absorbance against the control according to the following equation:

Scavenging activity%=[(Abs control-Abssample)/Abs control]×100

### Determination of total polyphenols

Total phenolic content of the extracts were determined colorimetrically, using the Folin–Ciocalteu method as described by Singleton et al. (1999). One milliliter of each of the sample extracts were added to 1 mL of Folin–Ciocalteu reagent followed by addition of 1 mL of an aqueous 7.5% solution of sodium carbonate. The mixture was shaken and allowed to stand for 30 min after which the absorbance was read at 765 nm. The results were expressed as milligrams of Gallic acid equivalents per gram powder (mg GAE/g powder) using the following standard curve line equation.

Y=0.031x+0.120

### Microbial analysis

#### Preparation of the inoculums

Isolates of the following organisms, bacteria *Escherichia coli*,* Staphylococcus aureus,* and *fungi Candida albicans* were obtained from Jomo Kenyatta University of Agriculture and Technology Food Microbiology Laboratory**.** The fungi were stored on sabouraud dextrose agar slants in the refrigerator at 4°C prior to use. Muller Hinton Agar was prepared and used according to the manufacturer's instructions. The cultures used were 24 h old, having been incubated at 37°C. The standard bacterial colonies were placed on the dry Muller Hinton Agar media and spread evenly on the plate and left to dry completely.

#### Preparation of paper disks

The paper disks used for bioassay were prepared from filter paper. The filter papers were punched to produce 6 mm paper disks. These disks were then sterilized at 15 lbs pressure at 121°C for 15 min in a well‐sealed universal bottle.

#### Impregnation of extracts onto paper disks for bioassay

Different concentrations between 0 and 100 percent of the crude extract in methanol were prepared. About 20 *μ*L of each dissolved fraction were applied on to a sterile paper disk and left to dry completely at room temperature for a few minutes. The dried paper disks were used for antimicrobial assay as described below.

#### Microbial susceptibility testing

##### Antibacterial assay

The extracts were tested for antibacterial activity using agar disk diffusion assay according to Shahidi et al. (2004). The strains of bacteria obtained were inoculated in test tubes containing nutrient broth and incubated at 37°C for 24 h (seeded broth). Media was prepared using Muller Hinton Agar, poured on Petri dishes and inoculated with the test organisms from the seeded broth. Sterile extract impregnated disks were picked by forceps and carefully placed on to the inoculated Muller Hinton Agar plates and incubated at 37°C for 24 h. Antibacterial activities were assayed by measuring the inhibition zone formed around the disks using a ruler in centimeters. The mean antibacterial activity was obtained from three replications (Junaid et al. 2006). The sterile distilled water and methanol served as negative control for aqueous and methanolic extracts, respectively, whereas standard antibiotic sensitivity disks containing eight antibiotics were used as standards or positive controls.

##### Antifungal activity assay

The antifungal activity assay was carried out using the disk diffusion agar method (Ajaiyeoba et al., 1998). Saboraud dextrose agar (SDA) and potato dextrose agar (PDA) were used using the procedures described above.

### Statistical analysis

All data were reported as mean (± standard error) of three replicates. Analyses of variance (ANOVA) were performed using SAS (Cary, NC, USA) portable version 9.1.3 release. Differences at *P* < 0.05 were considered significant. Treatment means found were separated using Duncan's Multiple Range Test.

## Results

Results of the proximate analysis of the mango seed kernels on dry matter basis are shown in Table 1. There were significant differences at *P* < 0.05 in the moisture content depending on the location. Due to this, the rest of the parameters were calculated based on dry matter basis. From the results, mango seed kernel powder contains on average, 6.74–9.20% protein content. Kent mango seed kernel from Makueni had the highest protein content of 9.20 while Apple mango seed kernel had the lowest protein content of 6.74. There were no significant differences in the protein content among the rest of the varieties from the two locations. Apple and Ngowe mango seed kernels had significantly higher fat content of 13.04 and 13.08, respectively, while Sabine from Makueni had the least fat content of 9.84%. The ash content ranged from 1.78 to 2.87%. There was a significant difference in the ash content of Ngowe from the two locations. The crude fiber ranged from 2.64 to 3.71%. There was no significant difference due to variety or due to locations in the fiber content. The carbohydrate content by difference ranged from 72.86 to 75.92%.

**Table 1:** Proximate composition of the mango kernel powders of the four mango varieties obtained from Makueni and Embu locations in Kenya

Variety	Location	Moisture Content	Crude Protein	Crude fat Content	Ash content	Crude fiber Content	Carbohydrates
Kent	Makueni	7.94 (±0.09)^a^	9.20 (±1.89)^a^	10.92 (±0.22)^bc^	2.44 (±0.16)^ab^	3.26 (±0.65)^a^	74.18 (0.70)^a^
	Embu	6.06 (±0.24)^b^	8.54 (±0.26)^ab^	12.32 (±0.08)^ab^	2.54 (±0.05)^a^	3.10 (±0.05)^a^	73.50 (0.22)^a^
Sabine	Makueni	7.25 (±0.34)^a^	8.44 (±0.84)^ab^	9.84 (±0.04)^c^	2.30 (±0.13)^ab^	3.71 (±0.61)^a^	75.71 (1.14)^a^
	Embu	5.70 (±0.15)^b^	7.19 (±0.27)^ab^	10.84 (±0.14)^bc^	2.77 (±0.05)^a^	3.28 (±0.02)^a^	75.92 (0.22)^a^
Apple	Makueni	7.06 (±0.25)^a^	6.74 (±0.89)^b^	13.04 (±0.12)^a^	2.87 (±0.24)^a^	2.64 (±0.35)^a^	74.71 (0.97)^a^
	Embu	5.92 (±0.11)^b^	7.30 (±0.06)^ab^	11.84 (±0.66)^ab^	2.35 (±0.02)^ab^	2.87 (±0.02)^a^	75.64 (0.65)^a^
Ngowe	Makueni	7.67 (±0.62)^a^	7.63 (±0.23)^ab^	13.08 (±0.14)^a^	1.78 (±0.23)^b^	3.14 (±0.35)^a^	74.37 (0.46)^a^
	Embu	5.58 (±0.03)^b^	8.84 (±0.20)^ab^	11.90 (±0.08)^ab^	2.89 (±0.02)^a^	3.51 (±0.04)^a^	72.86 (0.27)^a^
Grand Mean		6.61	7.99	11.72	2.49	3.19	74.61
LSD		0.9255	2.3172	1.7541	0.6913	1.761	3.245
% cv		4.90%	4.90%	4.90%	4.90%	4.90%	4.90%

Values are means of three replicates and those with similar letters within the same column are not significantly different based on Duncan's Multiple Range Test.

**Figure 1:**
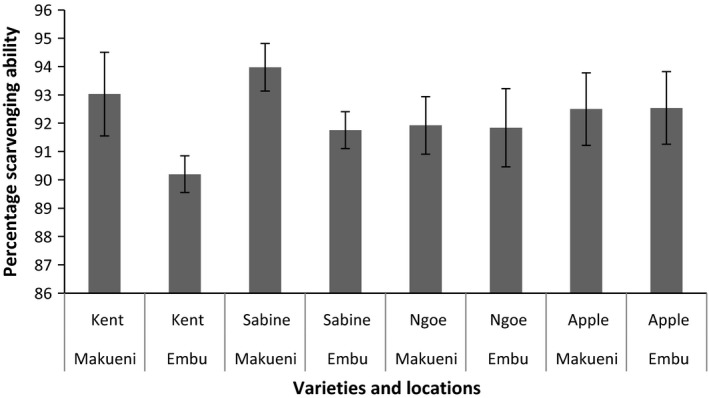
Percentage antioxidant capacity of the mango kernel extracts at 20 mg/mL. Percentage scavenging effect calculated from the decrease in absorbance at 517 nm against the control. Four mango varieties from Makueni and Embu counties. Results are replicates of three means (±SE). Error bars that do not cross each other represent values that are significantly different.

Results of the percentage scavenging ability of the mango seed kernel extracts are presented in Figure 1. The mean percentage scavenging ability of mango kernel extracts at the concentration of 20 mg/mL was 92.22%. There were significant differences in the location between Kent and Sabine mango kernel extracts. In both cases, Makueni presented significantly higher values. There were no significant differences due to location in Ngowe and Apple mango kernel extracts.

**Figure 2:**
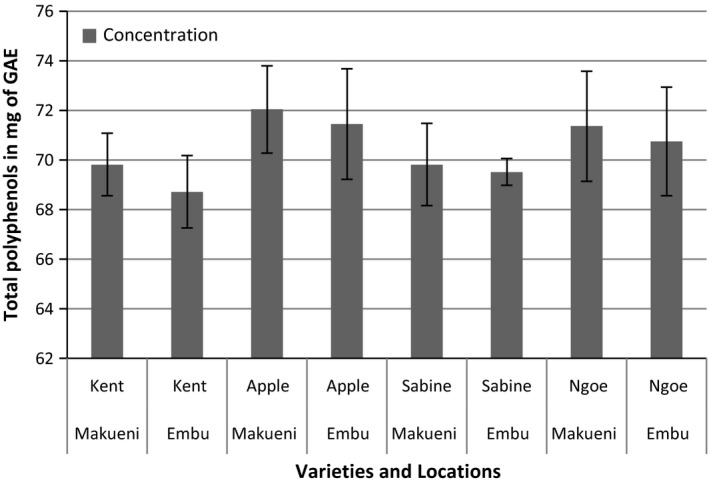
Total polyphenols in Milligrams of Gallic Acid Equivalent of the kernels from four mango varieties from Makueni and Embu counties. Absorbance read at 765 nm. Results are replicates of three means (±SE). Error bars that do not cross each other represent values that are significantly different.

As presented in Figure 2, the total polyphenols of mango kernel extracts ranged from 68.71 to 72.05. There were no significant differences attributed to either varieties or locations in the total polyphenols content.

The antimicrobial potential of the mango seed kernel extracts based on the inhibition zones against the specific microbes are presented in Table 2. The controls exhibited no inhibition while some of the antibiotic sensitivity disk and mango kernel extracts had some zones of inhibition when introduced to some specific microorganisms. There were no significant varietal differences in *S. aureas* at similar levels. However, there was a decrease in inhibition with the decrease in concentration. Norfloxacin at 10 *μ*g had an inhibition of 2.95 cm against *S. aureas* which is comparable to 2.07 cm inhibition by Ngowe and Kent mango kernel extracts at full concentration. Apple and Sabine mango kernel extracts at full concentration had significantly high inhibition zones of 1.93 and 1.73 compared to Kent and Ngoe with 1.13 and 1.10, respectively, against *E. coli*. Norfloxacin at 10 *μ*g had an inhibition zone of 2.95 cm against *E. coli* which is higher than that of Apple mango kernel extract. For *C. albicans,* the inhibition of Kent mango kernel extract at full concentration, 1.63, was significantly lower than that of Ngowe, Apple, and Sabine with 2.23, 2.13, and 1.83, respectively. On the other hand, the activities of clotrimazole (50 *μ*g), fluconazole (35 *μ*g), and nalidixic acid(30*μ*) were slightly higher than the inhibition of all the mango extracts on *C. albicans* Table 3.

**Table 2:** Zones of inhibition in centimeters by the extracts from the mango kernels. Means sharing the same superscript are not significantly different from each other at P<0.05)

	Concentration in percentage	*S. aureas*	*E. coli*	*C. albicans*
Apple	100	1.87 (±0.09)^ab^	1.93 (±0.09)^a^	2.13 (±0.12)^a^
	75	1.73 (±0.15)^ab^	1.27 (±0.03)^b^	1.53 (±0.09)^bcd^
	50	1.30 (±0.15)^bcde^	1.07 (±0.09)^bcd^	1.27 (±0.08)^cdefg^
	25	0.90 (±0.10)^e^	0.87 (±0.03)^cde^	1.06 (±0.09)^efgh^
Ngowe	100	2.07 (±0.18)^a^	1.10 (±0.06)^bcd^	2.23 (±0.09)^a^
	75	1.70 (±0.00)^ab^	0.97 (±0.12)^bcde^	1.50 (±0.11)^bcde^
	50	1.60 (±0.06)^abc^	0.80 (±0.05)^de^	1.13 (±0.12)^defgh^
	25	1.03 (±0.09)^cde^	0.83 (±0.07)^de^	0.97 (±0.09)^fgh^
Kent	100	2.07 (±0.15)^a^	1.13 (±0.03)^bcd^	1.63 (±0.08)^bc^
	75	1.53 (±0.09)^abcd^	0.87 (±0.12)^cde^	1.10 (±0.00)^defgh^
	50	1.27 (±0.09)^bcde^	1.03 (±0.12)^cbd^	0.87 (±0.03)^gh^
	25	0.93 (±0.09)^de^	0.63 (±0.03)^e^	0.73 (±0.03)^h^
Sabine	100	1.73 (±0.09)ab	1.73 (±0.12)^a^	1.83 (±0.09)^ab^
	75	1.33 (±0.20)^bcde^	1.23 (±0.03)^bc^	1.40 (±0.06)^bcdef^
	50	1.17 (±0.15)^cde^	1.07 (±0.03)^bcd^	1.33 (±0.09)^cdefg^
	25	0.80 (±0.06)^e^	0.83 (±0.03)^de^	1.03 (±0.09)^fgh^
LSD		0.6272	0.3981	0.4604
% CV		5.24%	5.24%	5.24%

Values >0.6 cm are an indicator of the real zones of inhibition.

**Table 3:** Inhibition zones of the controls**.** Values >0.6 cm are an indicator of the real zones of inhibition

Antibiotic	Concentration	Zones of inhibition in centimeters		
		*E. coli*	*S. aureas*	*C. albicans*
Ampicillin	25 *μ*g	0.80	0.85	0.00
Augmentin	30 *μ*g	0.75	0.00	0.00
Clotrimazole	50 *μ*g	0.95	0.00	2.90
Fluconazole	35 *μ*g	0.00	0.00	2.40
Gentamicin	10 *μ*g	1.85	1.95	0.00
Nalidixic Acid	30 *μ*g	1.55	1.70	2.50
Nitrofuranicin	50 *μ*g	0.85	0.90	0.00
Norfloxacin	10 *μ*g	2.95	2.25	0.00
Methanol	100%	0.00	0.00	0.00
Water	Sterile water	0.00	0.00	0.00

## Discussion

Varied medicinal properties are attributed to different parts of mango tree (leaves, fruit and bark) and these parts have been used as herbs in the Ayurvedic and indigenous medical systems for over 4000 years (Shah et al. 2010). This indicates that the mango plant can be a significant source of a range of biomolecules. However, there is virtually no commercial utilization of mango seed kernel which in most cases is discarded as waste in the fruit processing industry. This is despite the fact that mango seed tends to be between 20 and 60% of the whole fruit weight, and the kernel occupies between 45 and 75% of the seed depending on the variety (Maisuthisakul and Gordon 2009). Mango seed kernel oil has been reported to be a good source of polyunsaturated fatty acids such as oleic and linoleic acids which have health benefits (Kittiphoom and Sutasinee 2013). In this study, four mango varieties (Apple, Ngowe, Kent, and Sabine) from two mango‐growing regions in Kenya, Makueni, and Embu were analyzed for a number of biochemical and antimicrobial activity.

The Ngowe variety exhibited significant difference in the ash content subject to the production location (Table 1). This could be due to the fact that Makueni and Embu counties are two distinct agro‐ecological zones in Kenya. Embu County is semihumid and high potential area which lies at an altitude of 1200 m ASL with a mean annual temperature of 19°C and an annual rainfall stretching from 950 mm to 1350 mm. On the other side, Makueni County is a low potential area with an annual rainfall of less than 550 mm and a mean annual temperature ranging between 26°C and 35°C. The proximate analyses data on mango seed kernel from the four varieties is comparable to other reports by previous studies. For instance, studies have reported, 7.53% proteins, 11.45% fat, 2.20% fiber, and 1.0% ash content in dried mango kernel flour (Yatnatti et al., 2014), 13% crude oil, 6.36% crude proteins, 32.24% carbohydrate (by difference), 2.02% crude fiber, and 3.2% ash (Nzikou et al. 2010), and 5.90% moisture content, 5.20% crude protein, 76.14% carbohydrate, 9.84% fat, 0.49% crude fiber, and 2.43% ash (Orijajogun et al. 2014). Hence, the kernel powder of the mango varieties from the two locations in this study has more or less similar proximate composition to the other fruits in other regions.

**Figure 3:**
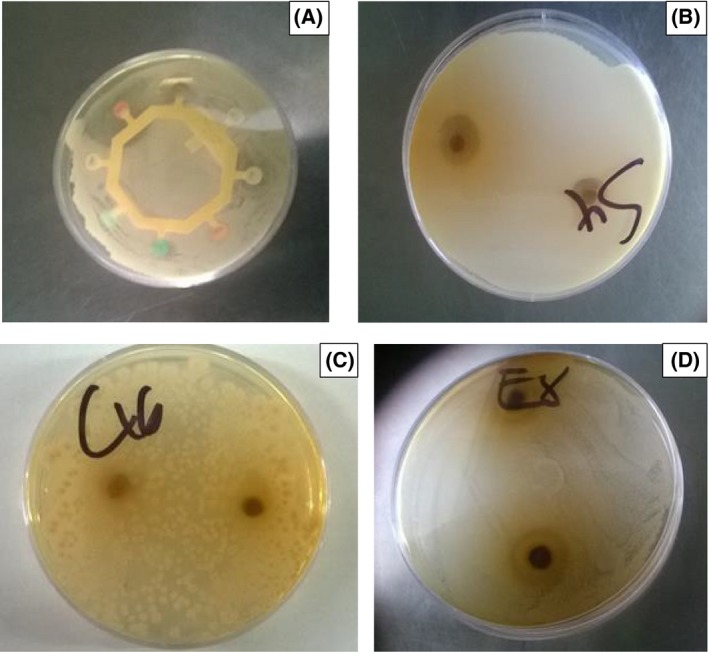
Images on inhibition zones. Picture 1, Inhibition zones by the standard sensitivity disks on *Staphylococcus aureas* plate. Picture 2, Inhibition zones by the 100% Kent mango kernel extracts on *Staphylococcus aureas*. Picture 3, Inhibition zones by the 25% Apple mango kernel extracts on *Candida albicans*. Picture 4, Inhibition zones by the 75% Sabine mango kernel extracts on *Escherichia coli*.

Mango kernel powder in this study was found to have high antioxidant capacity and total polyphenol contents (Figs. 2, 3). There were slight significant differences (*P* < 0.05) in the amount of total scavenging ability depending on the location between the Kent and Apple mango kernels. On the other hand, there were no significant differences in the total polyphenols depending on either variety or the locations. Ashoush and Gadallah (2011) reported DPPH scavenging ability values of 95.08% ± 0.10.

The potent antimicrobial activity demonstrated by the mango kernel extracts could be attributed to the presence of specific phytochemicals such as flavonoids, terpenes, tannins, and coumarins (Orijajogun et al. 2014). High antimicrobial and antifungal activity against *Staphylococcus aureus*,* Bacillus subtilis*,* Pseudomonas aeruginosa*,* Escherichia coli*, and *Candida albicans* has been reported in kernel powder of South African mango variety *(*Ahmed et al. 2005). The methanolic extracts in this study were found to have greater inhibition against *S. aureas* which is a gram‐positive bacterium at various concentrations than *E. coli* which is a gram‐negative bacterium. This can be explained by the structural difference in their cell walls. Both gram‐positive and gram‐negative bacteria are surrounded by a peptidoglycan cell wall and on top of this cell wall, the gram‐negative bacteria are surrounded by an exterior cell membrane made of lipopolysaccharides. This extra layer of lipopolysaccharides could be the one giving the gram‐negative bacteria more resistance to the antibiotics (Beveridge 1999). The results of this study are comparable to Kabuki et al. (2000) and Abdalla et al. (2007) who reported that mango kernel extracts exhibited antimicrobial activity against gram‐negative bacteria, for example, *E. coli* and *Salmonella spp* probably due to high content of different phenolic compounds, fatty acids, tocopherols, squalene, and different sterol components.

## Conclusion

The results from this study give strong credence to the utilization of the mango seed kernel as a potential natural antibiotic and antifungal to address the challenge of food poisoning and infections caused by pathogenic microorganisms. Such a natural compound has no issue with food safety as it is safe for human consumption. The major drawback in the commercialization of a plant extract as a natural antioxidant is the availability and cost‐effectiveness. However, since mango seed kernel is discarded as waste, the availability and cost factor will be negligible in generating a cheap and effective plant‐based natural antioxidants for the food industry.

## Conflict of Interest

None declared.
